# Azodicarbonamide, Hydrogen Peroxide, and l‐Ascorbic Acid Aid in the Modification of Protein Texture During High‐Moisture Meat Analog Extrusion Processing

**DOI:** 10.1111/1750-3841.70346

**Published:** 2025-07-03

**Authors:** Aniket Kamboj, Jana K. Richter, Chih‐Ling Lee, Joshua Bernin, Preston Watanabe, Jing Zhao, Brennan Smith, Girish M. Ganjyal

**Affiliations:** ^1^ School of Food Science Washington State University Pullman Washington USA; ^2^ School of Exercise and Nutritional Sciences San Diego State University San Diego California USA; ^3^ USDA ARS Food Processing and Sensory Quality Research New Orleans Louisiana USA

**Keywords:** functional additives, high‐moisture extrusion, microstructure, texture properties, wheat protein isolate

## Abstract

Protein texturization during high‐moisture extrusion is a complex process. Functional additives, like azodicarbonamide (ADA), hydrogen peroxide (H_2_O_2_), and l‐ascorbic acid (AA), play a critical role in modulating protein structure and texture during food processing applications. However, the specific effects of these additives on protein texture, microstructure, and functionality during high‐moisture meat analog (HMMA) extrusion remain underexplored. This study aimed to understand how these additives at different concentrations (0.1%–2.0%) influence the texturization of wheat protein isolate (WPI) during extrusion at 45% and 60% moisture levels. WPI was selected due to its well‐known reactivity and fibrous texturization capability. The extrudates were analyzed for their texture, microstructure, and physicochemical characteristics. Amino acid composition was determined using ninhydrin‐based ion‐exchange chromatography. At low concentrations, ADA, H_2_O_2_, and AA improved cross‐linking and aggregation within the protein matrix, increasing hardness, chewiness, and cutting force. However, higher concentrations disrupted the protein network, yielding more porous and fragmented structures, evident from the observed scanning electron microscopy (SEM) and confocal laser scanning microscopy (CLSM) micrographs. Color (L*, a*, b*) shifted significantly with increasing additive concentration, with ADA resulting in brighter and yellower samples, whereas AA and H_2_O_2_ had moderate effects. Amino acids mostly remained unchanged, but key residues, such as proline and lysine content, were slightly modified and correlated with the observed textural modifications. This study investigated how thermally reactive and oxidative additives influence protein cross‐linking and aggregation during extrusion, and how these molecular‐level changes govern fibrous texture formation in plant‐based meat analogs. Potential ADA and H_2_O_2_ residues post‐extrusion underscore the need for future safety‐focused quantification.

## 1 Introduction

The growing demand for sustainable, plant‐based food products has accelerated the development of high‐moisture meat analogs (HMMAs), which are designed to replicate the texture and sensory experience of animal meat (Dinali et al. 2024; Zhang et al. 2023). These meat analogs provide several environmental and health benefits, including reduced greenhouse gas emissions, decreased resource consumption, and potential nutritional advantages such as lower saturated fat content (Bryant 2022; Fresan et al. 2019; Shanmugam et al. 2023; van Vliet et al. 2020). The fact that meat substitutes still differ from meat in terms of texture, mouthfeel, flavor, taste, and nutritional value has, nevertheless, led to a small shift in population consumption so far (Hoek et al. 2011; Kendler et al. 2021; Szenderák et al. 2022).

Plant proteins, particularly wheat protein isolate (WPI), offer promising functionality for creating meat analogs due to their ability to form structured, fibrous textures through a process known as protein texturization (Maningat et al. 2022). During high‐moisture extrusion (HME), the texturization process transforms the molecular and structural properties of proteins, leading to the development of anisotropic (directionally aligned) textures that closely mimic the bite and chewiness of traditional meat products. This textural transformation is important not only for product appeal but also for overall sensory quality, affecting factors such as mouthfeel, juiciness, and tenderness.

A critical aspect of the texturization process is the formation of disulfide bonds between protein molecules, which serve as critical cross‐linking mechanisms that stabilize the protein network during extrusion (Richter, Smith, et al. 2024). The literature has widely acknowledged the importance of disulfide bond formation through the oxidation of sulfhydryl groups or disulfide interchange in achieving fibrous texture of meat analogs (Lee and Lanier 1995; Richter, Smith, et al. 2024; Schmid et al. 2022). Wheat protein contains glutenin and gliadin fractions, which are crucial for developing a meat‐like viscoelastic fibrous network. Gliadin contributes to viscous flow behavior, whereas glutenin provides elasticity and strength that enable wheat protein to form this fibrous structure (Webb et al. 2023). This is majorly achieved through the strategic reformation of disulfide bonds during texturization (Richter, Watanabe, et al. 2024). Previously, it has been established that adding reducing agents like cysteine and sodium metabisulfite during HME of wheat protein enhances protein cross‐linking and fibrous texture by facilitating early disulfide bond cleavage (Richter, Watanabe, et al. 2024). l‐Cysteine and l‐ascorbic acid (AA) are also reported to modify the fibrousness and mechanical properties of pea protein isolate and wheat gluten blends to enhance the textural attributes for improved meat analogs (Taghian Dinani et al. 2023). The reducing agents initially aid in splitting disulfide bonds and enabling protein strands to cross‐link during realignment, potentially improving fibrousness (Richter, Watanabe, et al. 2024; Schmid et al. 2022). Non‐covalent hydrogen bonding and covalent disulfide interactions are crucial for fibrous structure at higher moisture levels (∼50%). Additionally, the net effect of molecular interactions on meat analogs depends on process temperature, with the optimal range being 120–140°C for achieving directional alignment in cooling dies (Richter, Watanabe, et al. 2024; Taghian Dinani et al. 2023).

Although previous studies have examined the effects of reducing agents in modifying protein cross‐linking and textural properties, the impact of thermally reactive/oxidative functional additives on protein structuring during HME remains underexplored. In particular, the effects of additives such as azodicarbonamide (ADA), hydrogen peroxide (H_2_O_2_), and l‐AA on the protein network and textural development of WPI during extrusion are not yet well understood. Under extrusion conditions, these additives undergo chemical transformations that may impact protein structure. ADA, commonly used as a dough conditioner, decomposes thermally to release nitrogen and other by‐products, potentially influencing protein network formation by altering rheological behavior (La et al. 2004). H_2_O_2_, a strong oxidizing agent, can modify amino acid residues, thereby promoting disulfide bond formation and protein polymerization (Krämer et al. 2016). Though AA is typically a reducing agent, it oxidizes to dehydroascorbic acid under thermal processing. It can generate reactive oxygen species, contributing to protein cross‐linking and altering protein solubility and gelation behavior.

These thermally induced interactions are particularly relevant in the context of HME, where protein restructuring is key to developing meat analog textures (Aminlari and Majzoobi 2002; Nakamura and Kurata 1998). Despite their widespread use in other food applications, the specific contributions of ADA, H_2_O_2_, and AA to structural and functional transformations in WPI under extrusion conditions remain unclear. This study aims to fill that gap by systematically investigating their effects on the structural, textural, and morphological properties of WPI‐based meat analogs.

In brief, this study investigated the role of ADA, H_2_O_2_, and AA as protein modification agents in the texturization of WPI during HME. By examining their effects on disulfide bond formation, anisotropy, and other structural characteristics, we aim to gain deeper insights into the molecular mechanisms driving protein texturization. The findings of this research will contribute to a better understanding of how chemical modifiers can be strategically used to enhance the textural properties of meat analogs, ultimately improving their sensory and functional qualities.

## 2 Materials and Methods

### 2.1 Materials

Wheat protein (Arise 8000, WPI) was supplied by MGP Ingredients Inc. (Atchison, KS, USA). ADA 97.0% (1,1′‐Azobiscarbamide), sodium salt of l‐AA (C_6_H_7_NaO_6_), and hydrogen peroxide 30% (H_2_O_2_) were purchased from Sigma‐Aldrich Inc. (St. Louis, MO, USA), Biochemical Corporation (Cleveland, OH, USA), and Thermo Fisher Scientific Inc. (Fair Lawn, NJ, USA), respectively. All chemicals and reagents used were of analytical‐grade quality. WPI (6.38% moisture d.b.) contained 82.47% ± 0.13% protein, estimated using Rapid Max N exceed (Elementar Analysensysteme GmbH, Langenselbold, Germany) with an N_2_ conversion factor of 5.81 (Fujihara et al. 2008).

### 2.2 Preparation of Protein‐Additive Mixtures

WPI was blended with ADA or AA at concentrations of 0.1%, 0.5%, 1.0%, and 2.0% (w/w). The moisture content of the blends was adjusted to 10% ± 1% (w.b.) using a continuous spray of deionized water while mixing in a paddle mixer (Model A‐200, The Hobart Manufacturing Co., Troy, OH, USA). The mixtures were stored at 4°C overnight to ensure uniform moisture distribution. Before processing, the blends were allowed to come to room temperature (20°C ± 2°C). The desired H_2_O_2_ solution was introduced through the water port of the extruder, with the concentration adjusted so that the final H_2_O_2_ percentage [0.1%, 0.5%, 1.0%, and 2.0% (w/w)] was based on the weight of the dry protein feed.

### 2.3 Differential Scanning Calorimetry

Thermal analysis was conducted on unextruded blends of protein‐additive mixtures that were adjusted and conditioned to three distinct moisture contents: 10% (restricted moisture conditions), 50% (extrusion moisture levels), and 90% (excess moisture conditions). Approximately 10 mg of each sample was weighed and sealed in large‐volume capsules of O‐ring sealed, stainless steel (PerkinElmer Inc., Shelton, CT, USA). A differential scanning calorimeter (DSC 25, TA Instruments Inc., New Castle, USA) was used to measure the thermal transitions of the samples (Jeganathan et al. 2024). The temperature scanning range was set between 30°C and 250°C, with a heating rate of 10°C/min. This process enabled the determination of the thermal denaturation temperature (°C) and the enthalpy of transition (Δ*H*, J/g). Thermograms were generated using the instrument's proprietary software that came with the instrument (TA Instruments, New Castle, DE, USA). All thermograms were compiled and represented using OriginPro software (Version 2024b, OriginLab Corporation, Northampton, MA, USA) for comparative analysis.

### 2.4 Protein Texturization Using a Twin‐Screw Extruder Integrated With a Modular Cooling Die

**FIGURE 1:**
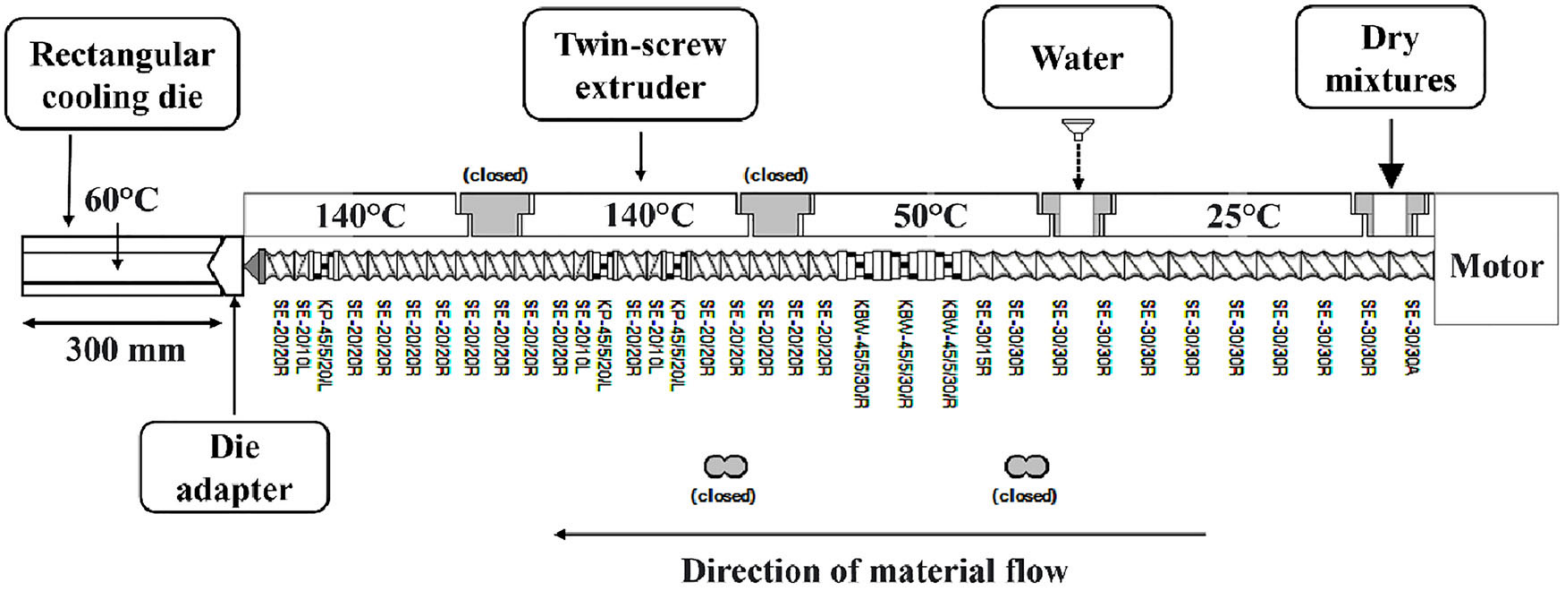
Twin‐screw extruder set‐up with screw profile designed for wheat protein extrusion in high‐moisture (∼50%) conditions with the attached rectangular cooling die of dimensions (30 cm × 25 mm × 7 mm). Screw elements used are SE‐20/20R, KP‐45/5/20L, SE‐2010L/R, SE‐30/30R, SE‐30/30A, and SE‐30/30R where codes define their design and function: SE indicates a standard screw element, KP refers to kneading paddles, the numbers (e.g., 20/20, 30/30) specify diameter and pitch, and R/L denotes right‐ or left‐handed orientation. Variations like A or angles (e.g., 45/5) indicate specific geometries or mixing tasks.

The extrusion trials were carried out using a co‐rotating twin‐screw extruder (Model TSE 20/40, 7.5 HP, Brabender GmbH & Co. KG, Duisburg, Germany) with a 40:1 length/diameter ratio with a screw diameter of 20 mm (Bernin et al. 2024). The screw profile was strategically designed to optimize the various stages of extrusion, including feeding, kneading, compression, and fiber formation. The first section of the screw comprised conveying elements (SE‐20/20R), which facilitated material feeding and hydration. These were followed by kneading elements (KP‐45/5/20L and SE‐2010L/R) that induced high shear to promote protein unfolding and mixing with the chemical modifiers. The central part of the screw contained alternating forward and reverse kneading elements (SE‐30/30R, SE‐30/30A) designed to enhance mixing and align protein fibers. The final segment consisted of conveying elements (SE‐30/30R) to ensure material transport into the cooling die, where fiber stabilization occurred. The detailed screw profile and twin‐screw extruder setup are shown in Figure 1. The cooling die had a modular design, with dimensions of 34 cm × 25 mm × 7 mm, allowing controlled extrudate cooling and facilitating fiber alignment and stabilization (Wagner et al. 2024). A constant screw speed of 300 rpm and a total feed rate of 80 g/min were maintained, with a temperature profile of 140°C in the melting and shearing zones and 60°C in the final half of the cooling die to facilitate fiber alignment and stabilization. The extrusion was done at two input moisture contents (45% and 60%) with ADA, H_2_O_2_, and AA at 0.1%, 0.5%, 1%, and 2% concentrations. This setup resulted in 24 unique extrusion trials (2 moisture levels × 3 modifiers × 4 concentrations). Each trial was duplicated to ensure reproducibility, leading to 48 samples. The specific mechanical energy (SME) was calculated using in‐line torque measurement data and screw speed values, considering the given moisture content and feed rate. SME values were determined as per (Kowalski et al. 2018), with each data point representing the average of 20 randomly recorded in‐line torque measurements.

### 2.5 Texture and Shear Profile Analysis

Texture analysis of the extrudates was performed using a Texture Analyzer (TA‐XT2i, Stable Micro Systems, Surrey, UK) following the previous literature (McClements et al. 2021; Zink et al. 2024). The extrudates were cut into uniform rectangular pieces (22 mm × 22 mm) prior to analysis. Briefly, the samples were compressed twice, with a pre‐test speed of 1.00 mm/s, a test speed of 1.00 mm/s, and a post‐test speed of 1.00 mm/s. The target mode was set to strain at 50%, with a 0.01 s interval between compressions. The hardness and chewiness values were obtained from the force‐time curves generated during the test using the following equation:

(1) $$\mathrm{Chewiness}\;=F_{\max}\times \left(\frac{A_{2}}{A_{1}}\right)\times d$$

where *F*_max_ is the maximum force from the first compression (hardness). *A*_1_ and *A*_2_ are the areas under the first and second compression curves, respectively. *d* represents the distance or height the sample recovers after the first compression. Cutting strength analysis was conducted to determine the horizontal and vertical cutting forces. The test mode was set with a pre‐test speed of 1.00 mm/s, a test speed of 1.00 mm/s, and a post‐test speed of 10.00 mm/s. The target mode was distance, set to 10.00 mm, and the trigger type was auto (force), with a trigger force of 0.10 N. The samples were cut fully, and the work done (N m) required for both horizontal and vertical cuts was recorded. Samples from each extrusion trial were analyzed 15 times individually (*n* = 15), and the average values were reported.

### 2.6 Colorimetry

The color of HMMA samples was measured using a spectrophotometer (CM‐5, Konica Minolta). Samples were prepared by evenly spreading them in a Petri dish to ensure a uniform surface. The instrument was calibrated using a white calibration plate (CM‐A210) and a zero‐calibration box (CM‐A124). An 8 mm target mask measured the sample in the L*, a*, b* color space. Twenty readings (*n* = 20) were taken across the sample surface to account for variability. The resulting L* (lightness), a* (red‐green), and b* (yellow‐blue) values were recorded for comparative analysis. The Δ*E* values were calculated to quantify the total color difference between the treated samples and their respective controls. For samples at 45% moisture, the reference was WPI without additives at 45% moisture, and similarly, for samples at 60% moisture, the reference was WPI without additives at 60% moisture. The Δ*E* values were determined using the following equation:

(2) $$\Delta E=\sqrt{{\left(L^{*}-L_{0}^{*}\right)}^{2}+{\left(a^{*}-a_{0}^{*}\right)}^{2}+{\left(b^{*}-b_{0}^{*}\right)}^{2}}\;$$

where $L_{0}^{*}$, $a_{0}^{*}$, and $b_{0}^{*}$, are the corresponding color values of the reference (WPI without additives).

### 2.7 Water‐Holding Capacity (WHC)

The extrudates were freeze‐dried and milled to make powder. The WHC of powdered extrudates was determined using the method described by Ahmedna et al. (1999). In brief, 1 g of the sample was suspended in 10 mL of distilled water in a centrifuge tube. The mixture was thoroughly dissolved using a rotary shaker and then centrifuged at 2000 × *g* for 10 min. The supernatant was discarded, and the percentage increase in the weight of the pellet was recorded as the WHC.

### 2.8 Scanning Electron Microscopy (SEM)

For SEM analysis, samples were fixed and dehydrated following standard procedures (Taghian Dinani et al. 2023). First, samples were cut to dimensions of approximately 5 mm × 5 mm × 3 mm. Primary fixation was done by immersing the samples in 2.5% glutaraldehyde aqueous solution and incubating for 8 h at room temperature at 4°C. After fixation, the samples were rinsed three times with distilled water for 5–10 min each. Dehydration was then performed through a graded ethanol series: 30%, 50%, 70%, 90%, and 100%, with each step lasting 10 min at room temperature. The final dehydration step in 100% ethanol was repeated three times for 10 min each. The samples were then dried using critical point drying (CPD). Following preparation, samples were mounted on SEM stubs and coated with a thin layer of gold using a sputter coater (208HRD, Cressington Scientific Instruments, Watford, UK) at a current of 20 mA for 60 s to ensure conductivity and prevent charging during SEM imaging. Surface morphology was examined using a Quanta 200F Field Emission SEM (FEI, Hillsboro, OR, USA) operated in high‐vacuum mode with an accelerating voltage of 20.0 kV and a working distance of approximately 13.2 mm. The chamber pressure was maintained at 130 Pa during imaging. Images were captured using an Everhart–Thornley detector (ETD) for secondary electron imaging at magnifications of approximately 2500× (10 µm scale) and 500× (50 µm scale) to analyze the microstructure of the samples.

### 2.9 Confocal Laser Scanning Microscopy (CLSM)

CLSM was employed to obtain information on the structural integrity and spatial distribution in the extrudates (Schreuders et al. 2021). The samples were sliced, and the middle layer was stained with Rhodamine B (0.1 mg/mL) to visualize the microstructure. Imaging was performed using a Leica SP8 CLSM (Leica Microsystems Inc., Deerfield, IL, United States) with a 560 nm excitation wavelength, and detection was done from 570 to 590 nm using HyD white light detectors. The laser power was set to 1%. Scanning was conducted in the *z*‐axis stacking mode, where the *z*‐length (or height) of the sample was scanned to a focal depth of 100 µm. Representative confocal micrographs of 2048 × 2048 pixels were acquired and assembled for comparative evaluation.

### 2.10 Amino Acid Analysis

Protein content was analyzed using the Dumas method, with aspartic acid serving as a standard with a conversion factor of 5.81 (Fujihara et al. 2008). Amino acid compositions of freeze‐dried samples were determined via the ninhydrin method using a Hitachi L‐8900 Amino Acid Analyzer. Samples (50 mg) were hydrolyzed with 6 N HCl and phenol in sealed, nitrogen‐purged borosilicate tubes at 110°C for 22 h. Taurine was added as an internal standard to calculate recovery. Hydrolyzates were filtered, dried, and reconstituted with pH 2.2 HCl. Amino acids were derivatized with ninhydrin, separated via ion‐exchange chromatography, and quantified using standard curves with adjustments for recovery.

### 2.11 Statistical Analysis

The effect of the different additives (ADA, H_2_O_2_, and AA) at varying concentrations (0.1%, 0.5%, 1.0%, and 2.0%) and two extrusion moisture content levels (45% and 60%) on the studied responses was evaluated. Unless otherwise stated, the experimental design ensured replication of at least three (*n* = 3) for each treatment response. The results were reported as mean ± standard deviation. The multiple comparisons of means were performed using Tukey's multiple comparison test in XLSTAT software (version 2024.2; Addinsoft, New York, USA).

## 3 Results and Discussion

### 3.1 Thermal Analysis

**FIGURE 2:**
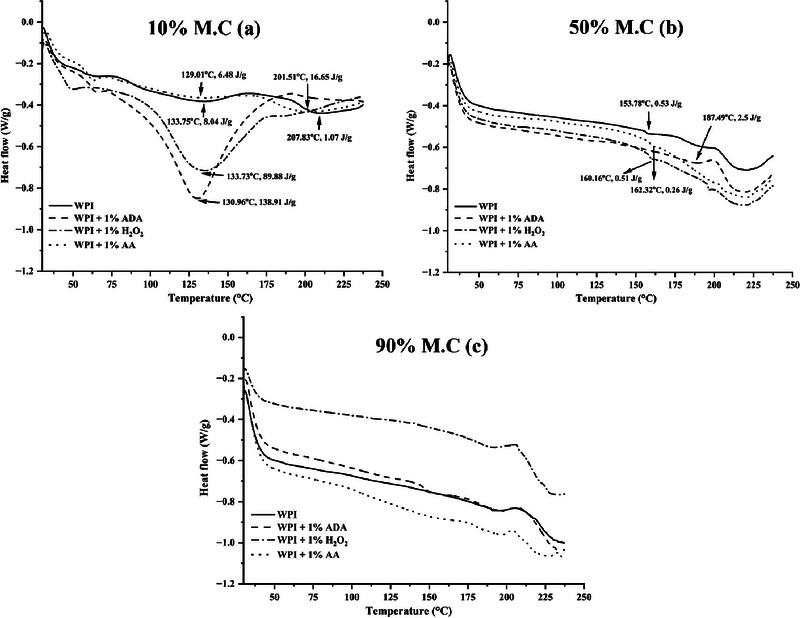
Differential scanning calorimetry (DSC) thermograms of wheat protein isolate (WPI) and WPI blended with 1% azodicarbonamide (ADA), 1% hydrogen peroxide (H_2_O_2_), and 1% l‐ascorbic acid (AA) at varying moisture contents: 10% M.C. (a), 50% M.C. (b), and 90% M.C. (c).

The DSC thermograms of WPI blended with ADA, H_2_O_2_, and AA at different moisture levels (10%, 50%, and 90%) are presented in Figure 2. At 10% moisture content (Figure 2a), the first endothermic peak appears around 55–65°C, likely due to the presence of small amounts of starch, as it aligns with its gelatinization temperature (Wang et al. 2021). Following this, two prominent endothermic peaks are observed for WPI at 133.75°C (enthalpy: 8.04 J/g) and 201.51°C (enthalpy: 16.65 J/g), indicating the characteristic thermal denaturation of wheat proteins. The values are consistent with those previously reported for WPI (Ahmed et al. 2008). The presence of two distinct peaks reflects the complex nature of wheat proteins, whose non‐homogeneous higher order structures unfold and refold at different stages during the heating process (Boire et al. 2013). The inclusion of ADA, H_2_O_2_, and AA impacts the denaturation temperatures and enthalpy values significantly. ADA showed a peak shift to 130.96°C with a sharp increase in enthalpy (138.91 J/g), suggesting that ADA induced structural changes that led to more energy‐intensive unfolding. Similarly, H_2_O_2_ elevated the enthalpy to 89.88 J/g at a peak temperature of 133.73°C, which might indicate enhanced protein cross‐linking. This might be attributed to the oxidative effects induced by H_2_O_2_ that helped prevent the further degradation of disulfide bonds that leads to higher peak denaturation temperature (Yousefi et al. 2024). AA caused a peak at 129.01°C with a relatively low enthalpy (6.48 J/g).

Furthermore, at 50% moisture content (Figure 2b), the DSC thermograms exhibit a different pattern with fewer distinct peaks, reflecting the plasticizing effect of water, which reduces the protein's thermal denaturation temperature, similar to what was reported by Jeganathan et al. (2024) for faba bean protein. For WPI alone, the major transition occurs at 153.78°C (0.53 J/g). The addition of ADA and H_2_O_2_ further increased the peak temperature (ADA: 187.49°C; H_2_O_2_: 160.16°C), indicating that these additives modified the thermal stability by interacting with the protein matrix. AA displays minimal impact on the thermal behavior, with a peak shift to 162.32°C but only slight changes in enthalpy. Under excess moisture conditions (90% moisture, Figure 2c), the thermal transitions become more subtle and less defined, reflecting the increased mobility of protein chains due to water plasticization (Jeganathan et al. 2024). Here, the additives exhibit minimal influence on peak temperatures, indicating that water dominates the thermal behavior. Although ADA and H_2_O_2_ still exhibit slightly higher enthalpy values than the control, the overall trends suggest that excess water interferes with chemical interactions between the additives and the protein matrix. The WPI‐H_2_O_2_ blend shows the highest residual heat flow, possibly due to oxidative cross‐linking or stabilization even at high water content.

### 3.2 Extrusion Process Response Parameters

**FIGURE 3:**
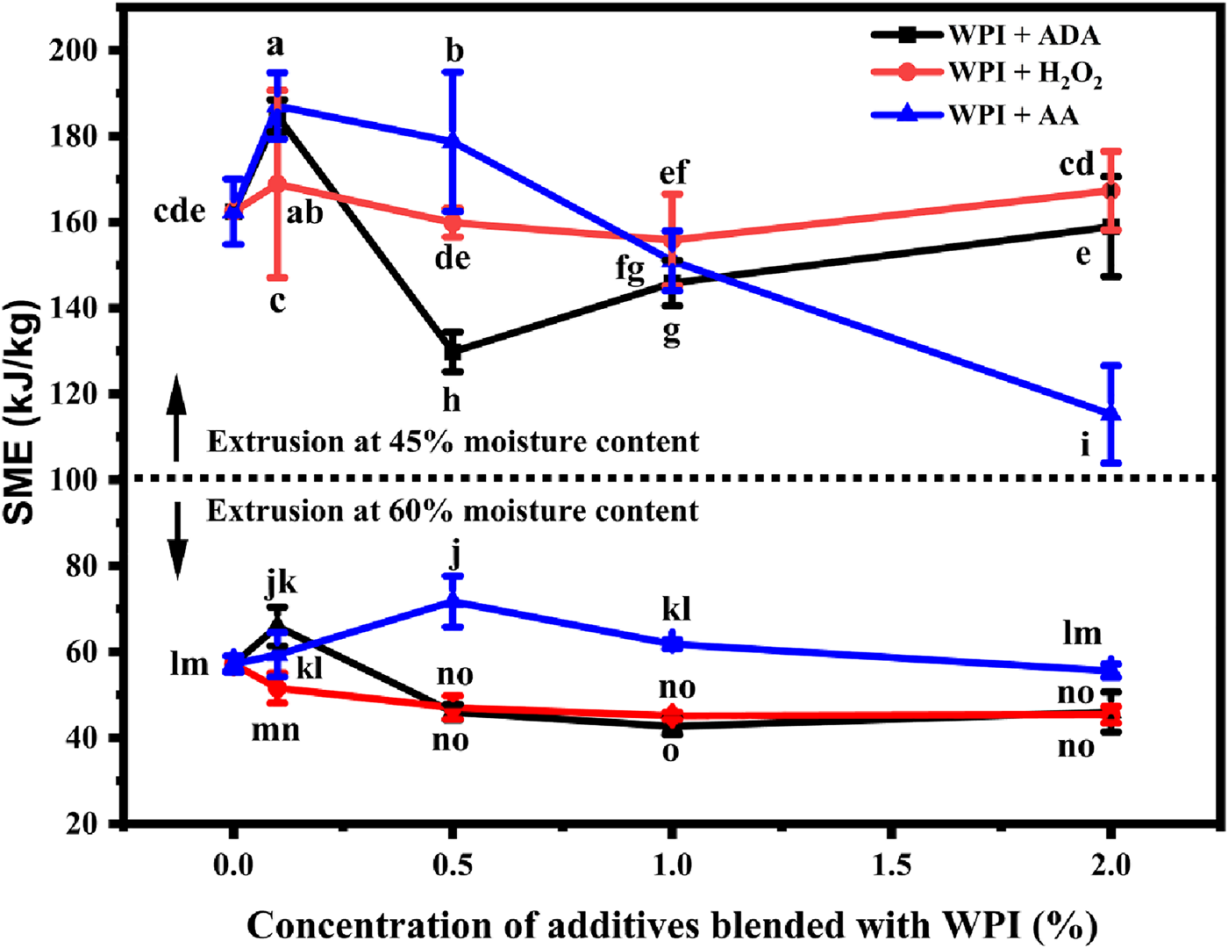
Effect of additive concentration and feed moisture content on the specific mechanical energy (SME) of WPI blends during extrusion. Three different additives were blended with WPI: azodicarbonamide (ADA), hydrogen peroxide (H_2_O_2_), and ascorbic acid (AA), represented by black, red, and blue lines, respectively. Data are presented as mean ± standard deviation. Different letters above the error bars represent significantly different values as determined by Tukey's multiple comparison test at 95% confidence level.

The SME values varied significantly across different moisture levels and additive concentrations (Figure 3). At 45% moisture content, SME was highest for WPI blended with 0.1% AA (187.05 kJ/kg), whereas the lowest SME was observed for 2.0% AA (115.20 kJ/kg). This trend suggests that lower concentrations of AA lead to higher energy requirements during extrusion, whereas higher concentrations reduce the mechanical energy demand. This is related to the dynamic viscosity of the material in the extruder barrel conditions (Morales Alvarez 2020). A similar pattern was observed for ADA and H_2_O_2_. However, ADA showed a distinct minimum SME at 0.5% concentration (129.77 kJ/kg), suggesting the interaction between ADA and WPI that might reduce the mechanical energy input required during the extrusion process. In other words, adding all additives led to larger viscosity dissipation in the extruder barrel beyond a certain minimum concentration. This might be attributed to the weakening of non‐covalent interactions, such as hydrogen bonding and hydrophobic interactions in proteins (Oswald et al. 2020). This leads to decreased protein–protein interactions, resulting in the partial unfolding or depolymerization of the protein network, which contributes to its lower viscosity. At 60% moisture content, the SME values dropped significantly compared to the samples that ran at 45% moisture content, reflecting the reduction in friction and resistance within the extruder, as higher moisture content leads to a more fluid system. The SME for WPI alone decreased from 162.41 kJ/kg (45% moisture content) to 57.15 kJ/kg (60% moisture content), and the further addition of additives decreased these values. The highest SME was observed with 0.5% AA (71.72 kJ/kg), whereas ADA at 1.0% and 2.0% led to some of the lowest SME values (42.70 and 46.05 kJ/kg, respectively) at 60% moisture content.

Back pressure is another important indicator of the resistance within the extruder, which can affect both the texturization of the product and overall extrusion efficiency (Ganjyal and Hanna 2004). At 45% moisture content, the addition of additives significantly affected the back pressure (Table 1). WPI alone exhibited a moderate back pressure of 285.23 psi, but this value dropped sharply for most treatments. For example, ADA at a concentration of 0.5% resulted in a substantial reduction in back pressure (147.83 psi), whereas H_2_O_2_ at a 2.0% concentration led to the lowest back pressure (114.70 psi). This indicates that certain additives, particularly ADA and H_2_O_2_, reduce the internal resistance within the extruder due to the enhanced flowability of the wheat protein blend. Interestingly, AA at lower concentrations had the opposite effect, causing a substantial increase in back pressure, with the highest pressure observed at a concentration of 0.5% (357.23 psi). This different behavior suggests that AA, especially at lower levels, may increase protein–protein interactions, leading to greater resistance during extrusion. The addition of AA in moderate amounts is known to substantially increase the hardness of wheat dough (Nakamura and Kurata 1997). However, at higher concentrations (2.0%), the back pressure dropped to 271.80 psi, implying a complex interaction where higher amounts of AA may lead to denaturation or modification of proteins that reduce system resistance. Consistent with the SME observations, back pressure values were notably lower across all samples at 60% moisture content. WPI alone exhibited a low back pressure of 107.47 psi, and the addition of additives further reduced these values. ADA at 1.0% and 2.0% showed the lowest back pressures (48.43 and 43.93 psi, respectively), indicating a significant reduction in internal friction. This trend was similar for H_2_O_2_ and AA, though the back pressure values for AA remained higher. This suggests that AA contributes to higher system resistance even at higher moisture levels, particularly at concentrations below 1.0%.

**TABLE 1:** Effect of moisture levels and chemical additives on the extrusion back pressure, color characteristics, and water‐holding capacity of texturized blends of WPI.

Extrusion moisture level (%)	Additives blended with WPI	Concentration (%)	Back pressure (psi)	L*	a*	b*	WHC (g/g)
45	WPI	—	285.23 ± 14.34^c^	46.37 ± 2.93^ijk^	0.82 ± 0.47^kl^	10.81 ± 0.74^m^	2.17 ± 0.05^def^
	ADA	0.1	172.97 ± 17.78^d^	43.53 ± 1.74^kl^	1.42 ± 0.25^ijk^	10.70 ± 0.82^m^	1.80 ± 0.09^ijk^
		0.5	147.83 ± 4.68^e^	60.43 ± 1.77^h^	6.11 ± 0.62^b^	24.13 ± 1.14^c^	1.78 ± 0.11^jk^
		1.0	171.57 ± 6.80^d^	61.59 ± 1.73^gh^	7.23 ± 0.52^a^	27.29 ± 0.93^b^	2.01 ± 0.08^fghij^
		2.0	151.50 ± 4.48^e^	64.44 ± 1.84^efg^	7.44 ± 0.65^a^	29.54 ± 0.93^a^	1.90 ± 0.07^ghijk^
	H_2_O_2_	0.1	326.67 ± 28.99^b^	46.02 ± 2.23^jkl^	0.87 ± 0.29^kl^	11.35 ± 1.43^m^	2.24 ± 0.06^bcd^
		0.5	147.47 ± 10.27^e^	42.91 ± 3.21^l^	1.11 ± 0.26^jkl^	10.60 ± 0.97^m^	2.06 ± 0.06^bcde^
		1.0	123.33 ± 7.59^fg^	47.00 ± 4.24^ij^	0.84 ± 0.38^kl^	14.18 ± 1.73^k^	2.08 ± 0.05^def^
		2.0	114.70 ± 7.84^gh^	49.48 ± 5.16^i^	1.26 ± 0.74^ijk^	17.85 ± 2.67^ij^	2.26 ± 0.03^def^
	AA	0.1	329.83 ± 31.46^b^	45.67 ± 3.29^jkl^	1.30 ± 0.53^ijk^	11.44 ± 1.20^lm^	2.37 ± 0.04^cdef^
		0.5	357.23 ± 7.26^a^	45.78 ± 3.22^jkl^	2.70 ± 1.97^ef^	14.06 ± 2.17^k^	2.36 ± 0.01^fgh^
		1.0	328.13 ± 7.96^b^	43.99 ± 3.40^jkli^	2.27 ± 1.22^efgh^	13.04 ± 1.28^kl^	2.19 ± 0.524^fg^
		2.0	271.80 ± 21.09^c^	49.79 ± 3.29^i^	3.68 ± 1.97^cd^	18.46 ± 3.70^i^	2.18 ± 0.04^cdef^
60	WPI	—	107.47 ± 3.31^hi^	71.28 ± 1.59^a^	1.61 ± 0.27^hijk^	18.76 ± 0.71^hi^	2.52 ± 0.14^ab^
	ADA	0.1	90.70 ± 9.85^j^	62.58 ± 0.85^fgh^	1.97 ± 0.20^fghi^	17.80 ± 0.89^ij^	2.04 ± 0.08^fghi^
		0.5	60.57 ± 35.40^k^	65.53 ± 2.44^cdef^	2.98 ± 0.18^de^	16.71 ± 0.89^j^	2.23 ± 0.03^cdef^
		1.0	48.43 ± 37.11^kl^	64.55 ± 3.02^efg^	3.89 ± 0.56^c^	20.20 ± 1.83^fgh^	1.71 ± 0.06^k^
		2.0	43.93 ± 26.33^l^	68.51 ± 1.81^abcd^	3.72 ± 0.35^cd^	21.39 ± 0.77^def^	1.82 ± 0.02^hijk^
	H_2_O_2_	0.1	121.10 ± 9.87^fgh^	68.68 ± 3.76^abcd^	1.79 ± 0.29^ghij^	19.09 ± 1.03^ghi^	2.14 ± 0.05^a^
		0.5	91.77 ± 9.38^j^	68.80 ± 2.84^abc^	1.96 ± 0.23^fghi^	20.79 ± 0.78^ef^	2.10 ± 0.18^ab^
		1.0	86.70 ± 3.26^j^	69.42 ± 5.32^ab^	0.86 ± 0.43^kl^	21.63 ± 0.86^def^	2.05 ± 0.03^a^
		2.0	85.13 ± 6.46^j^	71.79 ± 3.78^a^	0.36 ± 0.21^l^	22.03 ± 0.88^de^	2.12 ± 0.21^abc^
	AA	0.1	136.17 ± 4.01^ef^	68.87 ± 2.58^abc^	1.97 ± 0.40^fghi^	20.51 ± 0.67^efg^	2.14 ± 0.01^defg^
		0.5	145.70 ± 10.93^e^	65.23 ± 2.30^def^	2.47 ± 0.49^efg^	22.02 ± 1.12^de^	2.64 ± 0.03^fg^
		1.0	122.57 ± 2.28^fgh^	66.27 ± 1.08^bcde^	2.95 ± 0.50^de^	22.60 ± 1.01^cd^	2.60 ± 0.06^fghi^
		2.0	97.80 ± 1.81^ij^	63.54 ± 1.52^efgh^	3.71 ± 0.50^cd^	24.15 ± 1.21^c^	2.66 ± 0.01^efg^

Note: L*—lightness; a*—red‐green color axis; b*—yellow‐blue color axis. Data are presented as mean ± standard deviation (*n* = 30 for back pressure; *n* = 20 for L*, a*, b* values; *n* = 4 for WHC). Values within the same column followed by different superscript letters indicate a statistically significant difference at *p* < 0.05. The WHC value for non‐extruded WPI was 1.66 ± 0.01^k^ g/g. n/a indicates data not available.

Abbreviations: AA, ascorbic acid; ADA, azodicarbonamide; H_2_O_2_, hydrogen peroxide; WHC, water‐holding capacity; WPI, wheat protein isolate.

The SME and back pressure values demonstrated that ADA and H_2_O_2_ can significantly reduce energy consumption and internal friction during wheat protein extrusion. These findings are critical for setting up the extrusion process, as reduced SME and back pressure can translate into lower operational costs and improved product quality by minimizing degradation due to excessive mechanical forces (Thompson and Williams 2021). On the other hand, the use of AA, particularly at lower concentrations, requires control as it tends to increase back pressure, which can potentially affect the texturization and quality of the final extrudate.

### 3.3 Color Analysis

**FIGURE 4:**
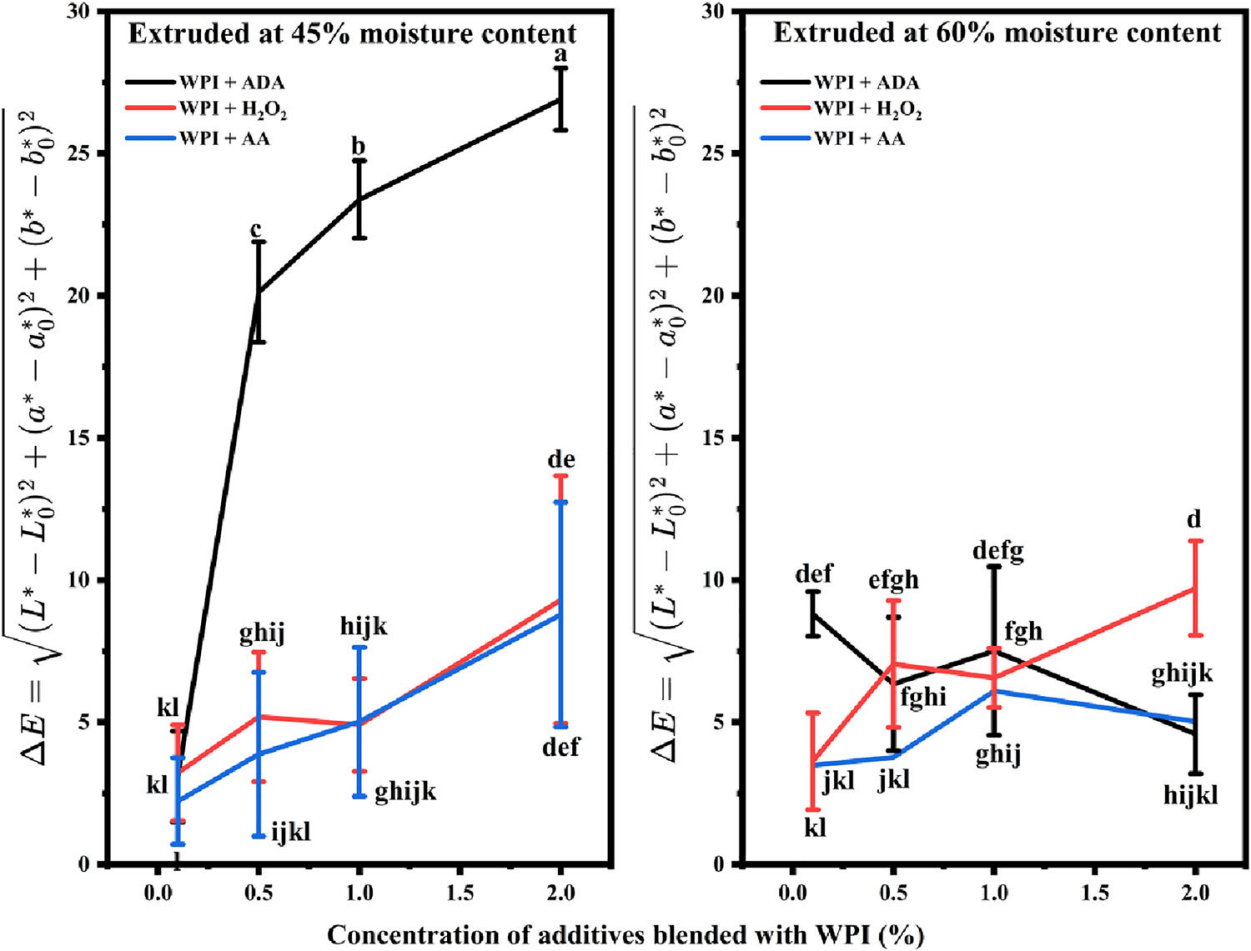
Effect of additive concentration and feed moisture content on the color difference (Δ*E*) of WPI blends during extrusion. The left panel represents samples extruded at 45% moisture content, whereas the right panel shows samples extruded at 60% moisture content. Three different additives were blended with WPI: azodicarbonamide (ADA), hydrogen peroxide (H_2_O_2_), and ascorbic acid (AA), represented by black, red, and blue lines, respectively. Δ*E* values were calculated on the basis of the color coordinates of the reference WPI extrudates: *L*₀* = 46.37, *a*₀* = 0.82, *b*₀* = 10.81 for 45% moisture and *L*₀* = 71.28, *a*₀* = 1.61, *b*₀* = 18.76 for 60% moisture. Data are presented as mean ± standard deviation. Different letters above the error bars indicate statistically significant differences (*p* < 0.05) as determined by Tukey's multiple comparison test.

The color analysis of WPI‐based HMMAs blended with additives revealed significant differences based on the type and concentration of the additive and the moisture content during extrusion (Table 1, Figure 4). At 45% moisture, WPI alone exhibited relatively low lightness (L* = 46.37 ± 2.93) and subtle red (a* = 0.82 ± 0.47) and yellow (b* = 10.81 ± 0.74) hues. The addition of ADA resulted in a marked increase in lightness at increasing concentrations (0.5%–2.0%), with L* values rising to 64.44 ± 1.84 and a corresponding increase in red (a* = 7.44 ± 0.65) and yellow (b* = 29.54 ± 0.93) tones, indicating a lighter and more vibrant product. H_2_O_2_ followed a similar pattern, where increasing concentrations resulted in higher L*, a*, and b* values, indicating a shift toward lighter, more colorful analogs. In contrast, AA produced different effects, particularly at lower concentrations (0.1%–1.0%), where the color remained darker, with L* values staying below 50. However, at higher concentrations (2.0%), the lightness increased to L* = 49.79 ± 3.29, accompanied by higher a* and b* values, indicating stronger red and yellow hues. This trend suggests that AA initially maintains a darker product but can induce lighter, more colorful tones at higher concentrations. At 60% moisture, the overall lightness of the extrudates increased across all samples. WPI alone at 60% moisture exhibited significantly higher lightness (L* = 71.28 ± 1.59), with moderate red and yellow tones (a* = 1.61 ± 0.27, b* = 18.76 ± 0.71). ADA and H_2_O_2_ treatments at this moisture level further enhanced lightness, with L* values reaching as high as 71.79 ± 3.78 for H_2_O_2_ (2%) and 68.51 ± 1.81 for ADA (2%) at higher concentrations. The a* and b* values similarly increased, leading to more pronounced red and yellow hues. H_2_O_2_ treatment at 60% moisture produced the lightest product (L* = 71.79 ± 3.78) with a relatively neutral red component (a* = 0.36 ± 0.21) and strong yellow hues (b* = 22.03 ± 0.88). Higher concentrations of ADA and H_2_O_2_ generally led to lighter and more colorful products, with pronounced red and yellow tones, whereas AA tended to maintain a darker color at lower concentrations, becoming lighter and more neutral at higher concentrations. These variations in color properties are important considerations for tailoring the appearance of meat analogs to meet consumer expectations.

### 3.4 Textural Properties and WHC

The results for the textural properties of the extrudates showed a significant impact of both moisture content and concentration of additives on hardness values (Table 2). At 45% moisture content, WPI samples exhibited a hardness of 29.55 ± 6.90 kg, which was significantly reduced when ADA was added. AA at lower concentrations (0.1%, 0.5%, and 1.0%) resulted in the highest hardness values, ranging from 33.58 ± 2.66 to 35.51 ± 3.88 kg, suggesting a strong contribution of AA to protein cross‐linking via disulfide bond formation (Gerrard et al. 2005). This is consistent with the role of AA in promoting cross‐linking reactions and enhancing the texturization of wheat protein (Nakamura and Kurata 1998). In contrast, ADA and H_2_O_2_, particularly at higher concentrations, generally reduced hardness at both moisture levels. For instance, at 2.0% ADA, hardness dropped significantly to 5.90 ± 1.37 kg. This reduction may indicate the excessive oxidation leading to over‐modification of the protein structure, weakening the gel matrix (Xiong and Guo 2020; Zhou et al. 2014). At 60% moisture content, the general trend observed was a reduction in hardness across all treatments compared to the 45% moisture level samples. This shows the effect of higher moisture contributed to a softer texture. WPI alone had a hardness of 10.34 ± 1.50 kg at 60% moisture, whereas adding additives further reduced it. However, the addition of AA still maintained relatively higher hardness values compared to ADA and H_2_O_2_ addition, which suggests its stronger role in maintaining firmness, even at higher moisture content.

**TABLE 2:** Mechanical and textural properties of high‐moisture meat analogs made from wheat protein isolate blended with different concentrations of azodicarbonamide (ADA), hydrogen peroxide (H_2_O_2_), and l‐ascorbic acid (AA) extruded at 45% and 60% moisture levels.

Additives blended with WPI	Concentration of additives	Hardness (kg)		Chewiness (N)		Horizontal cutting work (N m)		Vertical cutting work (N m)	
		45%	60%	45%	60%	45%	60%	45%	60%
WPI	—	29.55 ± 6.90^b^	10.34 ± 1.50^f^	237.77 ± 57.76^bc^	087.08 ± 11.90^f^	19.14 ± 1.99^bc^	6.42 ± 1.30^fg^	22.07 ± 2.19^ab^	8.70 ± 1.25^ghi^
ADA	0.1%	19.68 ± 2.88^c^	06.81 ± 0.82^gh^	145.54 ± 27.24^d^	052.09 ± 7.10^h^	20.33 ± 1.74^ab^	6.74 ± 0.55^f^	19.76 ± 2.19^c^	7.45 ± 0.79^hij^
	0.5%	12.25 ± 1.22^f^	03.60 ± 0.63^h^	085.54 ± 8.18^f^	024.00 ± 4.50^h^	09.70 ± 1.04^e^	3.80 ± 0.32^g^	10.69 ± 0.76^ef^	4.06 ± 1.05^j^
	1.0%	09.50 ± 2.46^f^	n/a	065.50 ± 17.81^fgh^	n/a	09.88 ± 0.69^e^	n/a	10.79 ±0.94ef	n/a
	2.0%	05.90 ± 1.37^gh^	n/a	042.22 ± 9.78^h^	n/a	09.75 ± 1.41^e^	n/a	09.32 ± 1.71^efgh^	n/a
H_2_O_2_	0.1%	34.50 ± 3.03^a^	06.94 ± 1.82^gh^	283.32 ± 25.78^a^	057.42 ± 14.77^gh^	19.31 ± 2.00^abc^	7.67 ± 1.45^ef^	22.82 ± 1.53^ab^	9.75 ± 1.04^efg^
	0.5%	18.22 ± 2.20^cd^	05.25 ± 0.72^h^	266.60 ± 16.60^de^	044.14 ± 5.14^h^	21.72 ± 2.32^ab^	7.93 ± 1.20^ef^	22.47 ± 1.31^ab^	8.42 ± 0.75^ghi^
	1.0%	19.46 ± 3.51^c^	07.10 ± 2.58^gh^	260.59 ± 23.77^de^	063.39 ± 13.06^fgh^	21.37 ± 3.32^ab^	8.18 ± 1.22^ef^	23.84 ± 2.46^a^	8.24 ± 1.30^ghi^
	2.0%	18.67 ± 2.34^cd^	07.23 ± 1.28^gh^	223.45 ± 14.62^de^	058.62 ± 11.68^gh^	21.94 ± 2.62^a^	7.16 ± 1.34^ef^	21.97 ± 2.05^b^	7.19 ± 1.69^ij^
AA	0.1%	35.51 ± 3.88^a^	15.57 ±1.18^de^	283.76 ± 32.22^a^	123.55 ± 9.76d^e^	16.77 ± 1.79^c^	5.72 ± 1.44^fg^	22.40 ± 2.05^ab^	9.55 ± 2.07^efg^
	0.5%	33.58 ± 2.66^a^	17.41 ± 1.48^cd^	141.91 ± 19.47^ab^	118.76 ± 26.73^e^	17.29 ± 2.76^c^	5.95 ± 1.14^fg^	23.39 ± 1.86^ab^	9.12 ± 0.62^efghi^
	1.0%	33.80 ± 2.62^a^	15.99 ± 1.03^d^	143.36 ± 28.06^ab^	120.86 ± 10.63^de^	21.81 ± 2.70^ab^	5.58 ± 1.18^fg^	23.62 ± 2.34^ab^	8.74 ± 0.96^fghi^
	2.0%	27.26 ± 4.88^b^	12.35 ± 1.25^ef^	137.17 ± 36.77^c^	081.24 ± 16.27^fg^	13.78 ± 3.89^d^	7.21 ± 1.57^ef^	17.64 ± 3.20^d^	8.50 ± 1.41^ghi^

*Note*: Data are presented as mean ± standard deviation (*n* = 30). Values for a given analysis at both moisture levels (45% and 60%) followed by different lowercase superscript letters indicate a statistically significant difference at *p* < 0.05. n/a indicates data not available.

The chewiness data complement the hardness data as chewiness was generally higher at 45% moisture content across all samples than those extruded at 60% (Table 2). This aligns with the expected textural softening at higher moisture levels, likely leading to a less cohesive and elastic structure. The trends in chewiness mirrored those seen in hardness. AA resulted in the highest chewiness values across both moisture contents, with chewiness reaching 283.76 ± 32.22 N at 45% moisture and 123.55 ± 9.76 N at 60% moisture for the 0.1% concentration. This suggests that AA facilitated more vigorous gel formation and firmer texture, likely due to enhanced protein cross‐linking (Mohammadi Nafchi et al. 2013). In contrast, adding ADA and H_2_O_2_ led to lower chewiness values, especially at higher concentrations. For instance, at 2.0% ADA, chewiness dropped to 42.22 ± 9.78 N at 45% moisture, which is consistent with its effect on hardness. This reduction in chewiness indicates the over‐processing of the protein network, leading to a softer, less cohesive texture.

The fibrous degree, which reflects the alignment and cohesiveness of fibers, can be estimated by taking the vertical and horizontal cutting work ratio. However, both forces are presented independently here to better understand their contributions to the texture. At 45% moisture content, the horizontal cutting work ranged from 9.70 ± 1.04 to 21.94 ± 2.62 N m, with the highest values observed for samples treated with AA and H_2_O_2_. Similarly, vertical cutting work at 45% moisture was highest for these samples, reaching up to 23.84 ± 2.46 N m for AA. This suggests that AA‐treated samples have a more cohesive, fibrous structure, contributing to higher resistance during cutting. As moisture content increased to 60%, horizontal and vertical cutting work decreased significantly, indicating a softer texture with reduced fibrous structure. For example, the WPI control at 60% moisture exhibited cutting work of 6.42 ± 1.30 N m (horizontal) and 8.70 ± 1.25 N m (vertical), compared to much higher values at 45% moisture. This reduction is consistent with the softening effect of higher moisture content, which disrupts the protein network and leads to a less fibrous, more amorphous texture.

WHC is critical in determining the moisture retention and overall texture of HMMAs. The WHC values (g/g) obtained in this study further elucidate the effect of moisture content and additive treatment on the final product quality. The WHC of non‐extruded WPI is approximately 1.66 g/g, consistent with values reported in the literature (Ahmedna et al. 1999). At 45% moisture content, WHC values for extruded and freeze‐dried milled products ranged from 1.78 ± 0.11 to 2.37 ± 0.04 g/g, with AA‐treated samples showing the highest WHC. Previous studies have also indicated that the WHC of hydrated WPI increases following heat treatment (Purwanti et al. 2013). These results suggest that AA enhanced the ability of the wheat protein matrix to retain water, likely due to stronger protein cross‐linking that results in a more cohesive structure capable of trapping moisture. This aligns with higher hardness and chewiness values, as a more structured protein network can hold more water and resist deformation during texture analysis. In contrast, ADA‐treated samples exhibited lower WHC values, particularly at higher concentrations (1.78 ± 0.11 g/g at 45% moisture for 1.0% ADA), corresponding to their lower hardness and chewiness. This suggests that ADA cross‐linking effect on protein structure may lead to reduced water retention capacity, likely due to the binding of hydrophilic sites in the protein network. At 60% moisture content, WHC values were generally higher across all samples, ranging from 1.71 ± 0.06 to 2.66 ± 0.01 g/g. Higher moisture availability likely contributes to an overall increase in water retention, though the textural properties (e.g., hardness, chewiness, and cutting forces) are softer due to the reduced cohesiveness of the protein network. Notably, the WHC of AA‐treated samples remained relatively high even at 60% moisture, again highlighting the strong cross‐linking effects. Higher WHC, particularly in AA‐treated samples, supports firmer, more cohesive textures, whereas lower WHC in ADA and H_2_O_2_ treatments corresponds to softer, less fibrous textures.

### 3.5 Macro and Microscopic Structure Analysis

**FIGURE 5:**
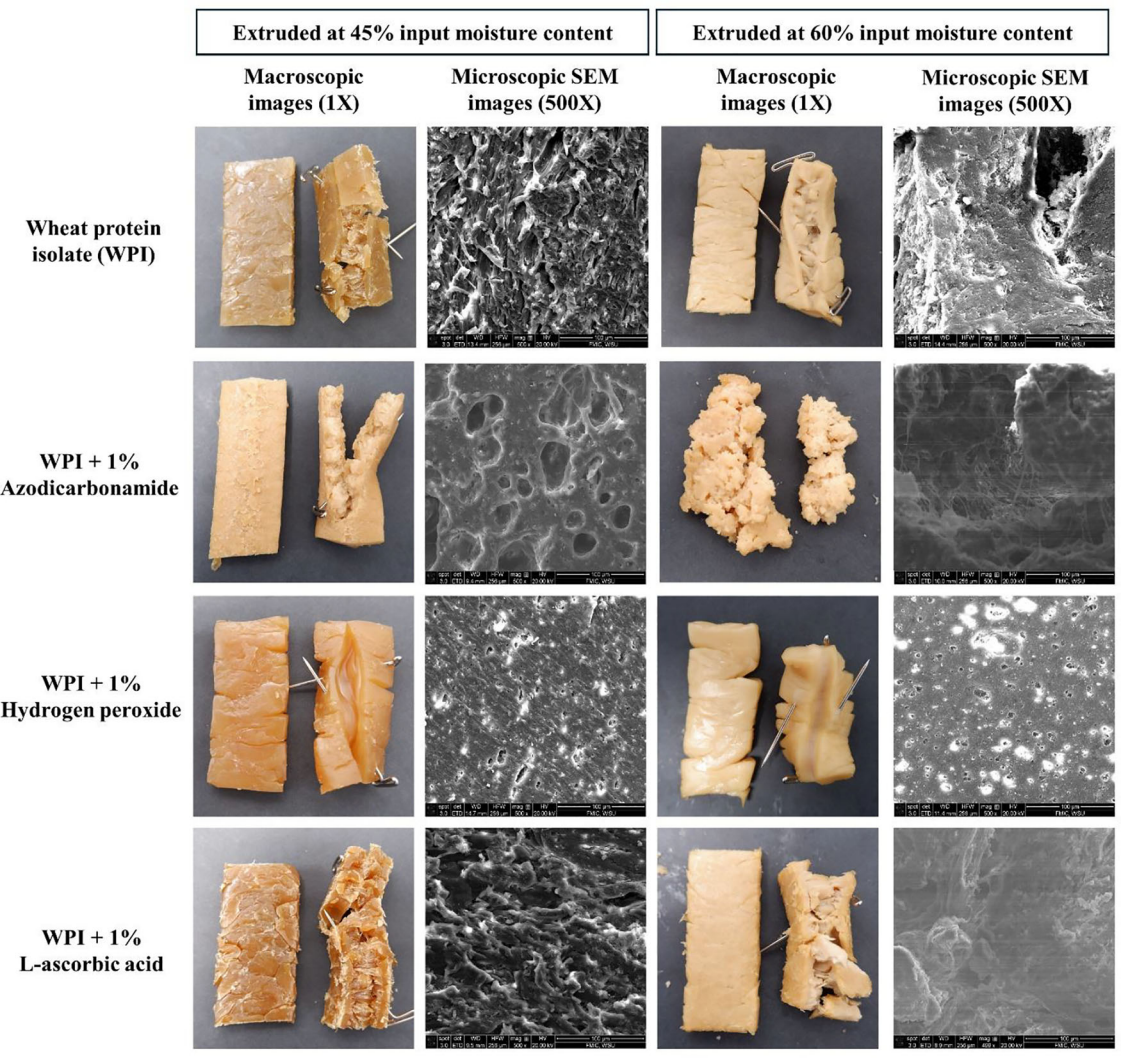
Macroscopic and microscopic SEM images of texturized wheat protein isolate (WPI) blends extruded at 45% and 60% moisture levels with and without additives. SEM, scanning electron microscopy.

The visualization of macroscopic and microscopic structures aligns closely with the TPA results. As observed in the hardness and chewiness data (Table 2), WPI extrudates treated with AA exhibited the highest values at 45% and 60% moisture content, correlating with the dense and fibrous microstructures in the SEM images (Figure 5). The tightly packed, aligned fibers observed in AA‐treated samples help explain the enhanced hardness and chewiness, as this type of protein network resists deformation and retains water more effectively, leading to firmer and more elastic textures (Li et al. 2018; Taghian Dinani et al. 2023). In contrast, the softer, less fibrous appearance of ADA‐ and H_2_O_2_‐treated samples corresponds to the lower hardness and chewiness values measured in the TPA. The fragmented, porous microstructures observed in the SEM images of these samples at both moisture levels reflect the reduced cohesiveness and weaker gel matrix, which leads to easier breakdown during texture analysis. ADA‐treated samples showed significantly lower hardness and chewiness, consistent with their highly porous and disrupted microstructure. These microstructural attributes correlate closely with (Ghanghas et al. 2024), who used nitrogen‐assisted HME of soy‐based meat analogs. Like ADA, nitrogen gas injection showed the production of micropores due to bubble formation. In this study, these micropores are most likely due to the thermal degradation of ADA into nitrogen and traces of carbon monoxide/dioxide (Antunes et al. 2010). Interestingly, ADA has shown potential for being used as an alternative to nitrogen injection during extrusion processing. The inability to maintain a dense protein network of samples containing ADA and H_2_O_2_ at higher moisture content, as seen in the SEM images, further supports the reduction in textural integrity and firmness. The cutting work data, which measures the horizontal and vertical forces needed to break the extrudates, also supports these observations. AA‐treated samples showed higher cutting forces, which can be attributed to the more aligned and cohesive structure observed microscopically, whereas ADA and H_2_O_2_ treatments showed lower cutting forces, reflecting the weaker and more porous structures. The SEM and macroscopic images provide a visual confirmation of the texture profile data, where the dense, fibrous structures of AA‐treated samples are linked to higher hardness, chewiness, and cutting forces. Meanwhile, the more fragmented and porous structures of ADA‐ and H_2_O_2_‐treated samples correspond to lower texture profile values, indicating weaker, softer products.

**FIGURE 6:**
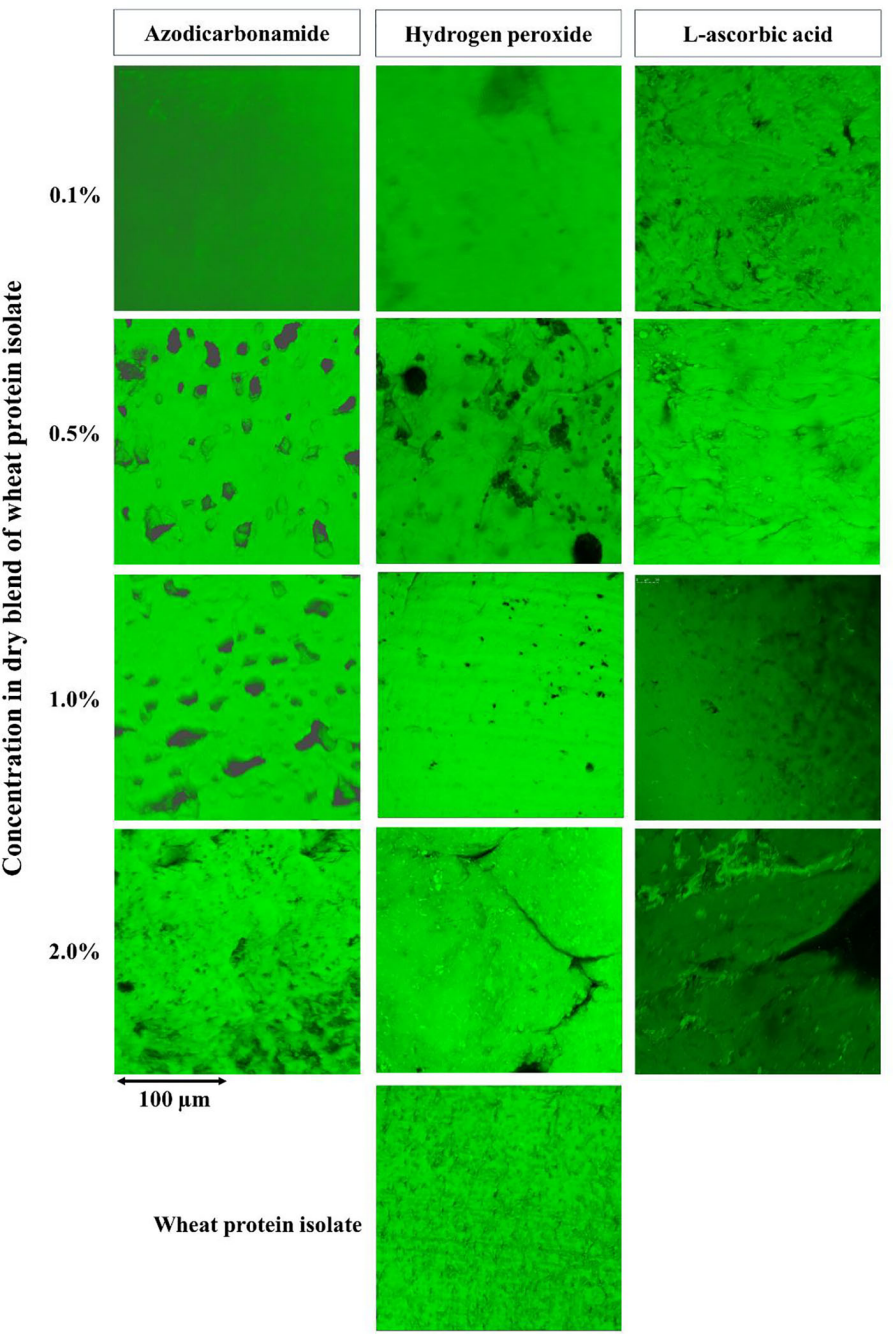
Confocal scanning laser microscopy images of wheat protein isolate‐based high‐moisture meat analogs extruded at 45% moisture content with different additives at various concentrations.

CLSM images provide detailed insights into the structural changes of WPI extruded at 45% moisture content with different concentrations of chemical additives (Figure 6). The control sample, without any additives, showed a dense and continuous protein network, indicating a well‐formed gel matrix in the absence of chemical modification. However, the introduction of additives caused notable changes in the protein structure. ADA, particularly at higher concentrations (0.5%–2.0%), significantly disrupted the protein matrix, leading to large void formations and a more porous, fragmented structure. This disruption aligns with the lower hardness and chewiness values observed in the TPA, as the broken matrix results in a softer, less cohesive product. H_2_O_2_ also weakened the WPI structure, though to a lesser extent than ADA. Although voids and fragmentation appeared at concentrations above 0.5%, the overall matrix was less fractured compared to ADA‐treated samples, suggesting a moderate impact on protein cross‐linking. Conversely, AA‐treated samples exhibited minimal disruption across all concentrations, with the protein matrix remaining dense and cohesive. At higher concentrations (1.0% and 2.0%), the network displayed well‐formed, elongated protein strands, reflecting the enhanced fibrous structure promoted by AA. These findings suggest the importance of additive selection in controlling the structural and textural properties of HMMAs. Although ADA and H_2_O_2_ reduce network integrity by introducing porosity and weakening the gel matrix, AA effectively stabilizes the protein structure, promoting a more fibrous and cohesive product. This structural behavior, observed at the microscopic level, complements the quantitative results from texture profile analysis.

### 3.6 Amino Acid Analysis

Freeze‐dried extruded samples showed slightly higher protein content (84.64% for 45% moisture and 83.46% for 60% moisture), with no significant changes observed in samples treated with H_2_O_2_ or AA (*p* > 0.05). ADA‐treated samples had increased nitrogen content due to their inherent non‐protein nitrogen, but total protein content remained unaffected, supported by consistent total amino acid levels. Amino acid compositions of freeze‐dried WPI samples treated with oxidizers and extruded at 45% and 60% moisture (Tables 3 and 4) showed no significant overall changes. Tryptophan and cysteine were not reported due to their destruction during acid hydrolysis. At 45% moisture, low H_2_O_2_ levels (0.1%–1%) increased Pro, with no significant effects on other amino acids. At 60% moisture, slight changes in Lys, Met, and Ser were observed. ADA reduced Lys, whereas 0.1% H_2_O_2_ increased Lys. High H_2_O_2_ levels decreased Met, but no sample differed significantly from the extruded WPI control. Ser decreased in extruded WPI (*p* < 0.05), but low H_2_O_2_ (0.1%–0.5%) and AA (0.1%–2%) reversed this effect.

**TABLE 3:** Amino acid compositions (g/100 g) of extruded wheat protein isolate samples with 45% moisture content modified with different levels of azodicarbonamide (ADA), hydrogen peroxide (H_2_O_2_), and l‐ascorbic acid (AA).

Amino acid	WPI (raw)	Extruded WPI	Azodicarbonamide (%)				H_2_O_2_ (%)				l‐Ascorbic acid (%)			
			0.1	0.5	1	2	0.1	0.5	1	2	0.1	0.5	1	2
**Ala**	1.27 ± 0.15	1.37 ± 0.45	1.27 ± 0.03	1.29 ± 0.06	1.26 ± 0.04	1.29 ± 0.11	1.26 ± 0.03	1.31 ± 0.00	1.30 ± 0.03	1.37 ± 0.03	1.46 ± 0.06	1.36 ± 0.02	1.41 ± 0.06	1.30 ± 0.05
**Arg**	1.69 ± 0.21	1.81 ± 0.59	1.68 ± 0.04	1.72 ± 0.08	1.67 ± 0.05	1.70 ± 0.14	1.78 ± 0.06	1.85 ± 0.03	1.84 ± 0.14	1.79 ± 0.09	1.94 ± 0.07	1.79 ± 0.01	1.89 ± 0.11	1.71 ± 0.07
**Asp**	1.50 ± 0.17	1.61 ± 0.53	1.49 ± 0.04	1.51 ± 0.07	1.47 ± 0.04	1.50 ± 0.12	1.60 ± 0.01	1.71 ± 0.01	1.62 ± 0.16	1.50 ± 0.07	1.67 ± 0.06	1.55 ± 0.01	1.63 ± 0.09	1.50 ± 0.06
**Glu**	17.66 ± 1.72	18.87 ± 6.61	17.43 ± 0.37	18.16 ± 0.67	17.40 ± 0.61	17.82 ± 1.53	20.73 ± 0.35	22.07 ± 0.05	20.45 ± 2.75	18.05 ± 0.38	18.89 ± 1.45	18.15 ± 0.10	18.18 ± 0.50	17.42 ± 0.57
**Gly**	1.78 ± 0.21	1.92 ± 0.64	1.77 ± 0.04	1.80 ± 0.08	1.75 ± 0.05	1.77 ± 0.15	1.82 ± 0.05	1.89 ± 0.00	1.85 ± 0.05	1.93 ± 0.05	2.03 ± 0.12	1.91 ± 0.03	1.96 ± 0.06	1.82 ± 0.06
**His**	1.02 ± 0.12	1.10 ± 0.37	1.00 ± 0.02	1.02 ± 0.04	0.99 ± 0.03	1.00 ± 0.08	1.12 ± 0.04	1.14 ± 0.00	1.07 ± 0.08	0.98 ± 0.03	1.15 ± 0.04	1.06 ± 0.01	1.11 ± 0.06	1.02 ± 0.04
**Ile**	1.65 ± 0.20	1.78 ± 0.59	1.62 ± 0.03	1.65 ± 0.08	1.61 ± 0.05	1.62 ± 0.13	1.73 ± 0.03	1.79 ± 0.00	1.77 ± 0.11	1.73 ± 0.08	1.89 ± 0.07	1.75 ± 0.02	1.84 ± 0.11	1.70 ± 0.06
**Leu**	3.14 ± 0.37	3.39 ± 1.13	3.11 ± 0.07	3.16 ± 0.15	3.08 ± 0.10	3.13 ± 0.26	3.19 ± 0.07	3.32 ± 0.01	3.30 ± 0.12	3.28 ± 0.15	3.60 ± 0.14	3.34 ± 0.04	3.52 ± 0.20	3.25 ± 0.14
**Lys**	0.78 ± 0.10	0.83 ± 0.28	0.76 ± 0.01	0.71 ± 0.03	0.62 ± 0.02	0.60 ± 0.05	0.87 ± 0.02	0.82 ± 0.01	0.85 ± 0.07	0.80 ± 0.04	0.87 ± 0.03	0.81 ± 0.01	0.86 ± 0.04	0.75 ± 0.03
**Met**	0.75 ± 0.08	0.81 ± 0.27	0.75 ± 0.02	0.76 ± 0.03	0.75 ± 0.02	0.74 ± 0.07	0.75 ± 0.01	0.79 ± 0.01	0.70 ± 0.03	0.59 ± 0.03	0.87 ± 0.03	0.80 ± 0.01	0.84 ± 0.05	0.77 ± 0.03
**Phe**	2.51 ± 0.29	2.70 ± 0.87	2.53 ± 0.05	2.59 ± 0.12	2.51 ± 0.08	2.61 ± 0.21	2.80 ± 0.07	2.88 ± 0.00	2.81 ± 0.21	2.65 ± 0.13	2.90 ± 0.10	2.69 ± 0.03	2.85 ± 0.17	2.63 ± 0.11
**Pro**	5.77 ± 0.71^d^	6.20 ± 2.01^cd^	5.72 ± 0.11^cd^	5.84 ± 0.27^cd^	5.70 ± 0.18^cd^	5.78 ± 0.46^cd^	8.97 ± 0.19^ab^	9.27 ± 0.01^a^	7.92 ± 2.17^abc^	5.71 ± 0.13^cd^	6.18 ± 0.29^bcd^	5.79 ± 0.07^cd^	6.01 ± 0.26^bcd^	5.59 ± 0.23^cd^
**Ser**	2.09 ± 0.09	2.12 ± 0.77	2.09 ± 0.11	2.09 ± 0.08	2.03 ± 0.07	2.08 ± 0.25	2.20 ± 0.13	2.44 ± 0.02	2.31 ± 0.25	2.20 ± 0.14	2.35 ± 0.05	2.23 ± 0.03	2.27 ± 0.16	2.00 ± 0.12
**Thr**	1.13 ± 0.12	1.19 ± 0.41	1.12 ± 0.03	1.14 ± 0.05	1.11±0.03	1.13 ± 0.11	1.22 ± 0.04	1.29 ± 0.00	1.23 ± 0.12	1.17 ± 0.05	1.28 ± 0.05	1.20 ± 0.01	1.24 ± 0.07	1.13 ± 0.05
**Tyr**	1.50 ± 0.20	1.63 ± 0.52	1.45 ± 0.04	1.48 ± 0.07	1.45 ± 0.06	1.45 ± 0.10	1.57 ± 0.02	1.56 ± 0.01	1.56 ± 0.09	1.51 ± 0.09	1.73 ± 0.06	1.61 ± 0.03	1.69 ± 0.10	1.59 ± 0.05
**Val**	1.84 ± 0.24	2.00 ± 0.67	1.81 ± 0.03	1.85 ± 0.09	1.81 ± 0.07	1.82 ± 0.15	1.94 ± 0.04	2.00 ± 0.01	1.96 ± 0.08	1.97 ± 0.07	2.15 ± 0.11	2.02 ± 0.04	2.08 ± 0.10	1.93 ± 0.08
**Total amino acids**	46.08 ± 4.21	49.32 ± 14.46	45.61 ± 0.75	46.75 ± 1.92	45.19 ± 1.42	43.46 ± 2.92	53.56 ± 1.16	56.12 ± 0.08	52.54 ± 6.37	47.24 ± 1.39	50.94 ± 2.71	48.05 ± 0.44	49.36 ± 1.90	46.10 ± 1.65

*Note*: Different lowercase superscript letters in the proline row indicate statistically significant differences at *p* < 0.05. No significant differences were observed for other amino acids. Data are presented as the mean ± SD.

**TABLE 4:** Amino acid compositions (g/100 g) of extruded wheat protein isolate samples with 60% moisture content modified with different levels of azodicarbonamide (ADA), hydrogen peroxide (H₂O₂), and l‐ascorbic acid (AA).

Amino acid	WPI (raw)	Extruded WPI	Azodicarbonamide (%)				H_2_O_2_ (%)				l‐Ascorbic acid (%)			
			0.1	0.5	1	2	0.1	0.5	0.1	0.5	1	0.5	1	2
**Ala**	1.27 ± 0.15	1.23 ± 0.04	1.18 ± 0.09	1.21 ± 0.06	1.28 ± 0.04	1.25 ± 0.03	1.35 ± 0.03	1.36 ± 0.04	1.32 ± 0.03	1.31 ± 0.03	1.34 ± 0.00	1.24 ± 0.08	1.33 ± 0.04	1.34 ± 0.04
**Arg**	1.69 ± 0.21	1.65 ± 0.05	1.58 ± 0.11	1.62 ± 0.07	1.70 ± 0.05	1.65 ± 0.04	1.81 ± 0.14	1.80 ± 0.04	1.72 ± 0.01	1.76 ± 0.02	1.77 ± 0.01	1.64 ± 0.12	1.75 ± 0.05	1.75 ± 0.06
**Asp**	1.50 ± 0.17	1.45 ± 0.04	1.39 ± 0.10	1.44 ± 0.06	1.50 ± 0.05	1.46 ± 0.03	1.58 ± 0.15	1.55 ± 0.04	1.47 ± 0.01	1.48 ± 0.02	1.58 ± 0.01	1.46 ± 0.10	1.56 ± 0.05	1.57 ± 0.05
**Glu**	17.66 ± 1.72	17.25 ± 0.64	16.52 ± 1.25	17.18 ± 0.78	17.67 ± 0.54	17.38 ± 0.35	19.33 ± 3.06	17.99 ± 0.79	17.48 ± 0.39	17.40 ± 0.47	18.55 ± 0.39	17.28 ± 1.10	18.67 ± 0.87	19.01 ± 0.61
**Gly**	1.78 ± 0.21	1.75 ± 0.05	1.66 ± 0.12	1.70 ± 0.08	1.79 ± 0.05	1.73 ± 0.04	1.92 ± 0.07	1.89 ± 0.06	1.85 ± 0.07	1.81 ± 0.04	1.88 ± 0.01	1.74 ± 0.12	1.86 ± 0.06	1.88 ± 0.06
**His**	1.02 ± 0.12	1.01 ± 0.03	0.94 ± 0.07	0.97 ± 0.05	1.02 ± 0.04	0.99 ± 0.02	1.08 ± 0.09	1.05 ± 0.02	1.00 ± 0.01	0.98 ± 0.01	1.05 ± 0.01	0.98 ± 0.08	1.04 ± 0.03	1.05 ± 0.04
**Ile**	1.65 ± 0.20	1.62 ± 0.04	1.53 ± 0.11	1.57 ± 0.07	1.64 ± 0.04	1.60 ± 0.04	1.74 ± 0.11	1.75 ± 0.03	1.67 ± 0.00	1.70 ± 0.02	1.71 ± 0.02	1.61 ± 0.13	1.72 ± 0.05	1.74 ± 0.06
**Leu**	3.14 ± 0.37	3.06 ± 0.09	2.93 ± 0.21	3.01 ± 0.14	3.15 ± 0.08	3.06 ± 0.07	3.30 ± 0.18	3.33 ± 0.07	3.19 ± 0.00	3.25 ± 0.03	3.24 ± 0.03	3.04 ± 0.22	3.26 ± 0.10	3.31 ± 0.11
**Lys**	0.78 ± 0.10^ab^	0.76 ± 0.02^ab^	0.71 ± 0.05^ab^	0.68 ± 0.03^b^	0.70 ± 0.02^ab^	0.66±0.01^b^	0.84 ± 0.09^a^	0.81 ± 0.01^ab^	0.77 ± 0.00^ab^	0.79 ± 0.01^ab^	0.79 ± 0.00^ab^	0.74 ± 0.05^ab^	0.79 ± 0.02^ab^	0.78 ± 0.03^ab^
**Met**	0.75 ± 0.08^a^	0.70 ± 0.03^ab^	0.70 ± 0.05^ab^	0.71 ± 0.03^ab^	0.75±0.01^a^	0.70 ± 0.02^ab^	0.77 ± 0.05^a^	0.73 ± 0.01^ab^	0.66 ± 0.00^ab^	0.60 ± 0.01^b^	0.80 ± 0.00^a^	0.73 ± 0.05^ab^	0.79 ± 0.03^a^	0.79 ± 0.02^a^
**Phe**	2.51 ± 0.29	2.40 ± 0.09	2.39 ± 0.17	2.45 ± 0.11	2.59 ± 0.07	2.54 ± 0.06	2.72 ± 0.27	2.68 ± 0.05	2.56 ± 0.01	2.63 ± 0.03	2.67 ± 0.02	2.51 ± 0.19	2.66 ± 0.08	2.69 ± 0.10
**Pro**	5.77 ± 0.71	5.65 ± 0.18	5.41 ± 0.39	5.55 ± 0.24	5.78 ± 0.19	5.62 ± 0.13	6.87 ± 2.24	5.75 ± 0.15	5.56 ± 0.09	5.55 ± 0.11	6.05 ± 0.05	5.67 ± 0.42	6.06 ± 0.20	6.14 ± 0.21
**Ser**	2.09 ± 0.09^abc^	1.92 ± 0.11^de^	1.87 ± 0.15^cde^	2.00 ± 0.14^abcde^	2.00 ± 0.15^abcde^	1.97 ± 0.03^bcde^	2.31 ± 0.23^a^	2.21 ± 0.19^ab^	2.15 ± 0.05^abcde^	2.10 ± 0.07^abcde^	2.25 ± 0.04^ab^	1.85 ± 0.05^e^	2.17 ± 0.14^abcd^	2.25 ± 0.03^ab^
**Thr**	1.13 ± 0.12	1.09 ± 0.03	1.04 ± 0.08	1.08 ± 0.05	1.12 ± 0.05	1.10 ± 0.02	1.21 ± 0.10	1.18 ± 0.04	1.15 ± 0.01	1.15 ± 0.02	1.18 ± 0.01	1.07 ± 0.05	1.18 ± 0.04	1.20 ± 0.04
**Tyr**	1.50 ± 0.20	1.47 ± 0.06	1.39 ± 0.10	1.42 ± 0.05	1.49 ± 0.02	1.43 ± 0.04	1.52 ± 0.09	1.55 ± 0.05	1.50 ± 0.02	1.56 ± 0.01	1.48 ± 0.02	1.47 ± 0.16	1.53 ± 0.05	1.55 ± 0.06
**Val**	1.84 ± 0.24	1.82 ± 0.04	1.71 ± 0.12	1.74 ± 0.08	1.84 ± 0.04	1.78 ± 0.04	1.97 ± 0.13	1.99 ± 0.02	1.91 ± 0.00	1.98 ± 0.03	1.92 ± 0.01	1.80 ± 0.13	1.92 ± 0.05	1.94 ± 0.08
**Total amino acids**	46.08 ± 4.21	44.83 ± 1.26	42.94 ± 3.16	44.33 ± 2.03	46.02 ± 1.41	44.92 ± 0.95	50.32 ± 7.00	47.620 ± 1.57	45.98 ± 0.61	46.05 ± 0.92	48.25 ± 0.61	44.85 ± 2.98	48.27 ± 1.84	49.01 ± 1.59

*Note*: Different lowercase superscript letters in the lysine, methionine, and serine rows indicate statistically significant differences at *p* < 0.05. No significant differences were observed for other amino acids. Data are presented as the mean ± SD.

### 3.7 Proposed Mechanistic Explanation

At lower concentrations (∼0.1%), ADA likely promotes cross‐linking between wheat protein molecules to some degree, most likely via disulfide bond formation and other covalent interactions, leading to a more rigid and compact protein matrix. This is evident from increased SME values (Figure 3), which indicate higher energy requirements during extrusion. The rigidity of the protein matrix can be related to the observed increase in proline content (Ghanaeian and Soheilifard 2018; Privalov et al. 1979). However, as ADA concentration increases (>0.5%), its oxidative effects might have disrupted the protein network (Beghin et al. 2022), breaking weaker bonds or forming misaligned cross‐links. This is reflected in decreased SME and back pressure, along with softer textures and lower hardness and chewiness values. At very high ADA concentrations (1.0% and 2.0%), the protein network becomes increasingly porous and fragmented, reducing WHC and weakening the gel matrix in terms of hardness. The observed reduction in lysine content at higher ADA levels supports this, as oxidation may preferentially target reactive amino acid residues (Utrera and Estévez 2012). At lower concentrations (∼0.1%), H_2_O_2_ acts as a mild oxidizing agent, facilitating limited protein aggregation, likely via disulfide bond formation, which explains the moderately higher SME and back pressure. However, as H_2_O_2_ concentrations increase (0.5%–1.0%), its stronger oxidative capacity begins to degrade weaker bonds in the protein network, reducing structural integrity. This is reflected in the stable but slightly decreasing SME values and lower back pressure, indicating less resistance to flow. At 2.0% H_2_O_2_, the protein network further distorted to a more porous, less cohesive structure with lower hardness, chewiness, and WHC values. The observed decrease in methionine (S‐containing amino acid) content at high H_2_O_2_ levels might be attributed to disrupting disulfide linkages critical for protein structure. AA seemed to promote cross‐linking of proteins, likely through disulfide bond formation (Taghian Dinani et al. 2023). AA enhances the protein network's cohesion at lower concentrations, as indicated by higher SME values and firmer textures with elevated hardness and chewiness. However, the formation of cross‐linkages might have resulted in an overly rigid and brittle protein matrix at higher concentrations (2.0%). Although higher AA concentration disrupts protein alignment, it generally maintains better textural integrity than ADA and H_2_O_2_. Although ADA and hydrogen peroxide (H_2_O_2_) are used here for their functional roles in protein structuring and oxidative modification, respectively, their potential residue levels post‐extrusion are of regulatory and safety interest. ADA undergoes thermal decomposition above 150°C, forming gaseous byproducts such as nitrogen, carbon monoxide/dioxide, and ammonia (Antunes et al. 2010), making its presence in the final product minimal. However, depending on extrusion conditions (e.g., short residence times and incomplete mixing), residual ADA or intermediate breakdown products could persist in trace amounts. Similarly, H_2_O_2_ decomposes readily under high temperature and mechanical shear, yet its residues can be detected using peroxidase‐based or chemiluminescent assays (Liu et al. 2010). Although direct residue measurements were not conducted in this study, future work should incorporate post‐processing quantification of both ADA and H_2_O_2_ to confirm compliance with regulatory thresholds and ensure food safety. This is especially relevant if the extrudates are intended for direct human consumption. This study explains how thermally reactive and oxidative additives affect protein cross‐linking and aggregation during HME. These changes at the molecular level influence the formation of fibrous structures and overall texture in plant‐based meat products. The findings can help manufacturers fine‐tune texture characteristics like firmness and chewiness by selecting appropriate additives and concentrations for specific product needs. Although this study focused on WPI, the insights gained regarding the influence of redox additives on protein network formation may be relevant to other plant proteins. However, the specific effects of ADA, hydrogen peroxide, and AA may vary due to differences in protein composition, especially in sulfhydryl content and aggregation behavior. Wheat protein was selected as a model due to its well‐known reactivity and fibrous texturization capability. Future work should explore how these mechanisms translate to other protein systems to broaden applicability in diverse plant‐based food products.

## 4 Conclusion

This study highlights the significant role of functional additives in modulating the texture and structural properties of WPI during HME. The results showed that ADA, hydrogen peroxide, and l‐AA impact product hardness, chewiness, color, and process efficiency through their microstructural effects attributed to protein cross‐linking and molecular interactions. Specifically, controlled ADA treatment can be a potential alternative to nitrogen gas injection in HMMA extrusion to produce porous structures in future studies. The extent of these effects is highly dependent on the concentration of these additives, moisture levels, and extrusion conditions, emphasizing the need for precise control during processing. Although this research advances the understanding of additive‐induced modifications in HMMAs, limitations remain regarding the detailed mechanisms underlying protein structural changes, such as the formation and rearrangement of disulfide bonds and other molecular interactions. Future studies should investigate these mechanisms at a molecular level to better elucidate the pathways involved. Additionally, exploring the interplay of additives with other ingredients, such as lipids or polysaccharides, could further enhance the understanding of the underlying mechanism and design of meat analogs. These findings lay the groundwork for more systematic approaches to improving plant‐based meat technologies through targeted chemical interactions.

## Author Contributions

**Aniket Kamboj**: conceptualization, formal analysis, investigation, methodology, visualization, data curation, writing ‐ original draft, writing ‐ review and editing. **Jana K. Richter**: conceptualization, investigation, visualization, data curation, methodology, formal analysis. **Chih‐Ling Lee**: data curation, formal analysis, writing ‐ review and editing. **Joshua Bernin**: data curation, methodology, formal analysis, writing ‐ review and editing. **Preston Watanabe**: data curation, formal analysis, methodology, writing ‐ review and editing. **Jing Zhao**: methodology, data curation, writing ‐ review and editing, formal analysis. **Brennan Smith**: methodology, formal analysis, writing ‐ review and editing, data curation. **Girish M. Ganjyal**: conceptualization, methodology, writing ‐ review and editing, project administration, visualization, funding acquisition, resources, supervision.

## Conflicts of Interest

The authors declare no conflicts of interest.
